# Superior target genes and pathways for RNAi‐mediated pest control revealed by genome‐wide analysis in the beetle *Tribolium castaneum*

**DOI:** 10.1002/ps.8505

**Published:** 2024-11-05

**Authors:** Benjamin Buer, Jürgen Dönitz, Martin Milner, Sonja Mehlhorn, Claudia Hinners, Janna Siemanowski‐Hrach, Julia K. Ulrich, Daniela Großmann, Doga Cedden, Ralf Nauen, Sven Geibel, Gregor Bucher

**Affiliations:** ^1^ Crop Science Division, Bayer AG, R&D, Pest Control Monheim Germany; ^2^ Department of Evolutionary Developmental Genetics University of Göttingen, Johann‐Friedrich‐Blumenbach Institute, GZMB Göttingen Germany; ^3^ Department of Medical Bioinformatics University Medical Center Göttingen Göttingen Germany

**Keywords:** RNAi, target genes, genome‐wide screen, *Tribolium castaneum*, *Phaedon cochleariae*, *Leptinotarsa decemlineata*

## Abstract

**BACKGROUND:**

An increasing human population, the emergence of resistances against pesticides and their potential impact on the environment call for the development of new eco‐friendly pest control strategies. RNA interference (RNAi)‐based pesticides have emerged as a new option with the first products entering the market. Essentially, double‐stranded RNAs targeting essential genes of pests are either expressed in the plants or sprayed on their surface. Upon feeding, pests mount an RNAi response and die. However, it has remained unclear whether RNAi‐based insecticides should target the same pathways as classic pesticides or whether the different mode‐of‐action would favor other processes. Moreover, there is no consensus on the best genes to be targeted.

**RESULTS:**

We performed a genome‐wide screen in the red flour beetle to identify 905 RNAi target genes. Based on a validation screen and clustering, we identified the 192 most effective target genes in that species. The transfer to oral application in other beetle pests revealed a list of 34 superior target genes, which are an excellent starting point for application in other pests. Gene ontology (GO) and Kyoto encyclopedia of genes and genomes (KEGG) analyses of our genome‐wide dataset revealed that genes with high efficacy belonged mainly to basic cellular processes such as gene expression and protein homeostasis – processes not targeted by classic insecticides.

**CONCLUSION:**

Our work revealed the best target genes and target processes for RNAi‐based pest control and we propose a procedure to transfer our short list of superior target genes to other pests. © 2024 The Author(s). *Pest Management Science* published by John Wiley & Sons Ltd on behalf of Society of Chemical Industry.

## Graphical abstract

RNAi based pest control is species‐specific and eco‐friendly but the optimal target genes have remained unclear. By a genome‐wide screen in the red flour beetle we identified the best target processes and showed transferability to other pests. The presented 34 *superior target genes* will guide applications in other species.

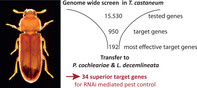

## 1 INTRODUCTION

The human population continues to grow and will reach over 9 billion by 2050 resulting in a growing demand in food supply. Together with a changing regulatory landscape and the threat of new resistances, novel crop protection solutions are required that are efficacious, durable, eco‐friendly and safe to nontarget organisms. RNA interference (RNAi)‐based solutions promise to offer such sustainable solutions with a different mode‐of‐action (MoA). However, despite many years of research there is no consensus, which genes or pathways are the best targets for RNAi‐mediated pest control.

RNAi is a naturally occurring defense mechanism against viruses and transposons, initially discovered in plants and in *Caenorhabditis elegans*, that has been intensively studied in various organisms. Essentially, introduction of dsRNA leads to a cellular response that destroys transcripts with sequence complementarity.^1^, ^2^ RNAi has become a valuable tool for gene function studies in many arthropods and it has been tested as new tool for species‐specific and eco‐friendly pest control. For instance, introduction of dsRNA targeting essential insect genes into plants has resulted in protection against pests such as the Western corn rootworm (WCR) *Diabrotica virgifera* and the Cotton bollworm *Helicoverpa armigera*,^3^, ^4^, ^5^ and to resistance of papaya to the Papaya Ringspot Virus.^6^ Variations include expression of dsRNAs in plastids^7^ and dsRNA molecules sprayed onto plant surfaces. Advances in dsRNA production systems have reduced the cost to less than US$0.5 g^−1^ where ≈0.3–4.9 g ha^−1^ are required in the field.^8^ Indeed, first sprayable RNAi products are entering the market targeting Colorado potato beetle (*Leptinotarsa decemlineata*)^9^ and guidelines on biosafety have been formulated.^10^

Notably, the efficacy of RNAi after oral uptake widely differs across species. An efficient response has been demonstrated for several coleopteran species including *D*. *virgifera virgifera* ^4^, ^7^, ^11^ the red flour beetle *Tribolium castaneum* ^12^, ^13^ and the mustard beetle *Phaedon cochleariae*.^11^ Translation to a number of other insect species including important lepidopteran pests, however, has failed or led to inconsistent results, e.g.^14^, ^15^ Limitations are thought to be related to uptake and metabolism of dsRNA rather than to a general malfunction of the RNAi mechanism.^16^ It has been speculated that a strong RNAi response is largely governed by efficient cellular uptake of dsRNA^17^, ^18^, ^19^, ^20^ and is prohibited by a high level of dsRNA‐degrading enzymes in the midgut and/or the hemolymph.^21^, ^22^, ^23^, ^24^, ^25^, ^26^ To overcome these challenges, new technologies for improvement of dsRNA delivery and stability are being explored.^27^, ^28^, ^29^

One of the key parameters for efficacy of RNAi‐mediated pest control is the choice of the target genes. So far, that choice has been inspired mainly by the results from few seminal studies, which had tested a limited number of genes, e.g.,^4^ were inspired by classic insecticidal targets or were derived from physiological knowledge, e.g.^5^ However, it has remained unclear whether – considering the different MoA of RNAi – one should target different biological processes compared to the pathways known from classic chemical insecticides. Much of this uncertainty can be overcome by unbiased large‐scale screening. Indeed, a previous RNAi screen in the red flour beetle *T. castaneum* revealed novel target genes, which showed higher efficacy compared to previously used target genes^13^, ^30^ and they were successfully tested in other species, e.g.^15^, ^31^ Furthermore, it identified the proteasome as an unexpected target process,^13^ and indeed a proteasome component is now being used for the first sprayable dsRNA application on the market.^9^

A genome‐wide analysis requires an appropriate laboratory model system where the red flour beetle *T*. *castaneum* is one of the most highly developed genetic model systems, and shows a strong and systemic RNAi.^17^, ^18^, ^19^, ^32^ Furthermore, it is a representative of Coleoptera, which is a clade containing a number of economically relevant pests that are amenable to oral dsRNA delivery. Importantly, *T*. *castaneum* had been established for genome‐wide screening before.^13^, ^30^, ^33^ Some reports indicated that RNAi delivery by feeding works in *T*. *castaneum*, but we and others have had no success in that respect.^15^

In order to gain a genome‐wide view on target genes and processes, we first performed a primary RNAi screen for lethality of 10 193 genes, which together with the previous screen covering 5337 genes,^13^, ^30^ adds to an almost genome‐wide coverage of 15 530 genes. The increased throughput was achieved by forgoing the morphological analyses and focusing on the lethality. Based on the genome‐wide screen, we defined a list of 905 *target genes* (top 5.8%). Gene ontology (GO) and Kyoto encyclopedia of genes and genomes (KEGG) analyses revealed that most of them belonged to basic cellular processes, which are not typical targets for classic insecticides. Dose–response validation of 807 of the *very good targets* and subsequent cluster analysis revealed 192 *most effective target genes* in the red flour beetle postinjection of dsRNA. We then tested a subset of 66 genes by oral feeding in another beetle pest species (mustard beetle) leading to a list of 34 *superior target genes*. These *superior target genes* were well‐transferable to another pest species (Colorado potato beetle). In summary, we reveal that RNAi‐mediated pest control should target biological processes different from chemical pesticides and we provide a list of genes, which represents an excellent starting point for identification of efficient RNAi target genes in other species, especially coleopteran pests.

## 2 MATERIALS AND METHODS

### 2.1 Primary screen

In this work, we screened 10 193 genes, that were not previously screened in Ulrich *et al*.^13^ to reach an almost genome‐wide coverage of 15 530 genes in *T. castaneum*. The screening followed the procedure extensively described in Schmitt‐Engel *et al*.^30^ with minor modifications. In brief, pBA19 L6 or L5 instar larvae were injected and scored for lethality. dsRNA solution at a 1 μg μL^−1^ concentration was injected into 10 randomly selected animals per experiment (mixed males and females). Per screening day, injections for 40 different genes and controls were performed, where the first round of injection of each day represented the negative control (injection buffer). No experiment was excluded. Note that in previous experiments, no adverse effects of nontargeting dsRNA injection on viability had been found.^17^, ^30^ Furthermore, the many genes of our screen that showed no phenotype despite dsRNA injection served as additional controls for dsRNA quality. Injections were performed 4 days a week with Day 5 (D5) being required for stock maintenance and documentation. Using this schedule, each week 156 novel genes were injected. On D7 and D16 postinjection, the lethality of the larvae, pupae and adults, respectively, was determined. The person analyzing the experiments had no knowledge on the respective gene IDs. In this study, we did not perform morphological analyses that were performed in the previous screen^30^ to increase the throughout and reach a genome‐wide coverage (see Supporting information Figs S1 and S5 for detailed information). For some months during the 2 years of screening, we encountered increased background lethality in our negative controls (daily buffer injections). This correlated with a concurrent infection of the beetles. Our analyses showed that using a cutoff of 100% lethality would almost completely remove false positive data. Importantly, during our follow‐up experiments with the best candidate genes, any false positive gene could be removed with confidence.

### 2.2 Functional annotation of genes

Functional annotation of *T*. *castaneum* genes was performed as described previously.^34^ In brief, Blast2go v1.3.3^35^ was used to summarize annotation of protein domain predictions from interproscan v5.17‐56.0^36^ and similarity searches against Uniprot KB using NCBI‐blastp v2.2.27.^37^

Functional and pathway enrichment analysis was performed using hypergeometric distribution with R package *goseq* v1.28.0^38^ with default parameters. For enrichment of pathways, annotation from the KEGG database was used.^39^

For visualization and clustering of enriched GO terms, REVIGO v1.8.1^40^ was used with default parameters. Clustering of GO terms was performed at a cutoff value of 0.9.

Clusteringk‐means clustering with R v3.6.2. The number (*n* = 5) of clusters was defined using the elbow method. A number of genes had not been screened with 300 ng μL^−1^ concentration. In order to include them in the analysis, we replaced these values with the values from the 30 ng μL^−1^ concentration. Based on our previous distribution and correlation analyses [Fig. 2(A),(B)] this should not introduce a concerning bias that would interfere with our aims.

## 3 RESULTS

### 3.1 A very high‐throughput organismal RNAi screen to detect essential genes

**Figure 1:**
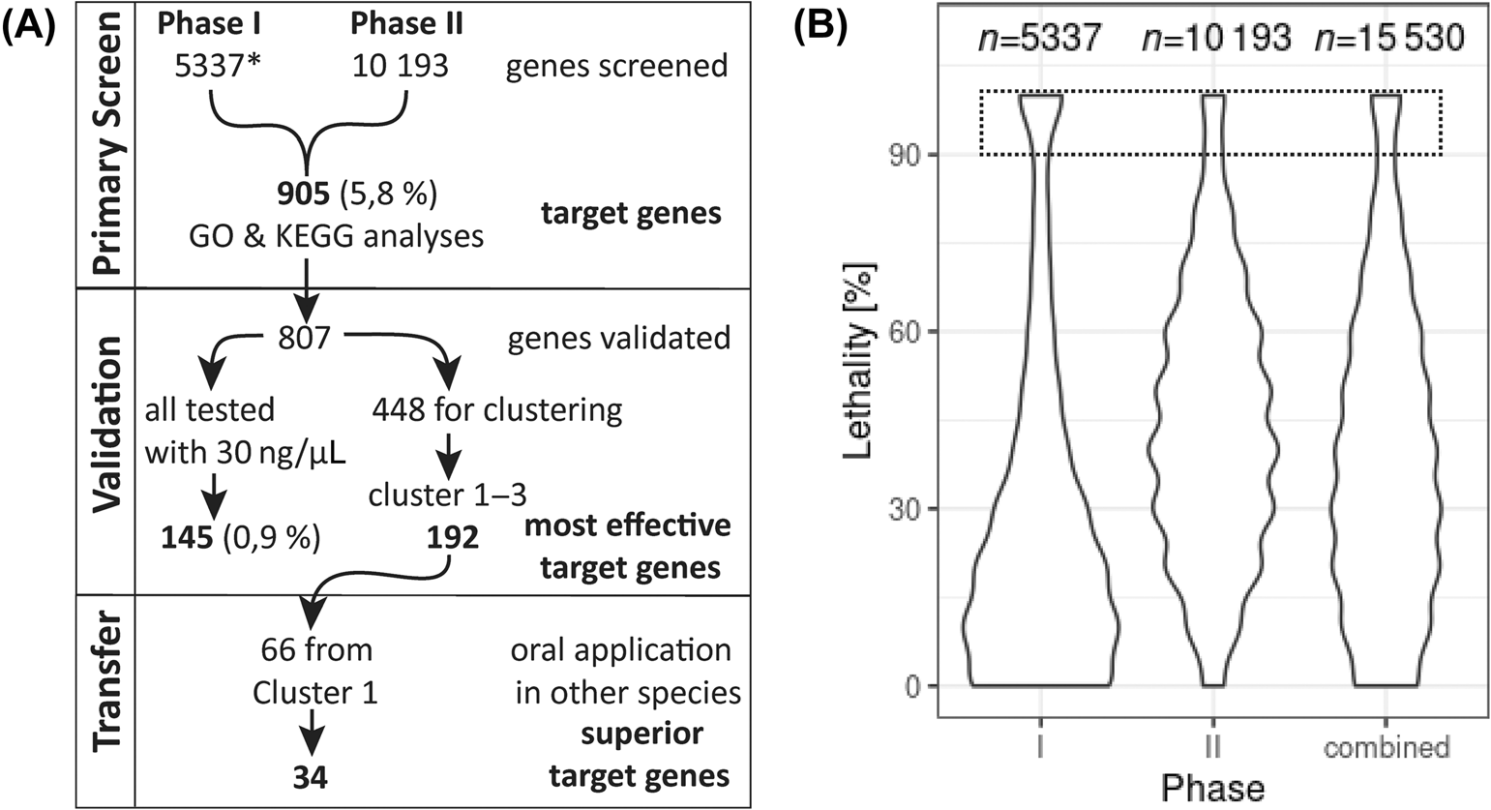
Overview and controls. (A) Flowchart with the different screening phases and analyses performed in this publication. *, genes analyzed by Ulrich *et al*.^13^ (B) Distribution of the lethality observed in phases I and II of the screen, and for the entire dataset. The width of the shapes reflects the portion of the experiments that led to a given percentage of dead animals (*n* = 10 injected larvae) 11 days postinjection (Phase I) or 7 days postinjection (Phase II). Datasets with nine or 10 dead (90% or 100% lethality; comprised in dotted box) were selected for the validation screen. Phase I: *n* = 5337 genes; Phase II: *n* = 10 193 genes; total *n* = 15 530 genes.

For a high‐throughput genome‐wide RNAi screen, a model species is required that reproduces all year round in the laboratory with unlimited access to animals. Furthermore, the dsRNA application needs to be extremely robust to reduce technical variability. We chose the red flour beetle *T*. *castaneum* for the high‐throughput part because it had been established for large‐scale RNAi screening via injection^12^, ^30^, ^33^ and RNAi delivery by feeding has not worked in our hands. Using that screening platform, we aimed at identifying a manageable number of genes to be further tested subsequently by oral feeding in other pest species. For the high‐throughput part, we opted for a two‐phase screening strategy where in the primary high‐throughput screen we tested all genes using one concentration of dsRNA and assessing lethality at one point in time, similar to the approach in a previous screen by Ulrich *et al*.^13^ In the subsequent validation screen (described in the next chapter), a selection of the *target genes* was tested further using different concentrations of dsRNA and scoring lethality several times after dsRNA injection [see Fig. 1(A) for an overview]. The first 5337 genes had been screened previously as part of the iBeetle screen (Phase I)^30^ and most of them had already been analyzed for potency as target genes for RNAi‐mediated pest control.^13^

With this work (Phase II), we scored another 10 193 genes using the same concentration and the larval stages but analyzing lethality already at D7 postinjection. The best RNAi target genes reliably induce death within a week in *T*. *castaneum* such that we did not expect to miss important target genes.^13^ While Phase I had a throughput of 25 genes per week per screener (including controls and extensive phenotypic annotations^30^), we reached a throughput of 156 genes per week per screener in Phase II (owing to lacking morphological analyses; see schedule and experimental details in Fig. S1). Based on our experience with several RNAi screens of different complexity,^13^, ^30^, ^33^ we think that this throughput is close to the upper limit for a large‐scale organismic RNAi screen in *T*. *castaneum* and probably for most if not all insects.

The distribution of the lethality found for each gene differed somewhat between phases of the screen [Figs 1(B) and S2] where in Phase II a lower portion of genes showed 90% lethality or higher [compare width of distributions within the box with broken outline in Fig. 1(B)]. This might reflect the fact that Phase I of the screen was biased towards more conserved and highly expressed genes, leading to an enrichment of basic cell biological processes. Furthermore, an increased overall level of lethality was observed for Phase II [Fig. 1(B); see also Fig. S2]. We assign the latter to an increased technical background lethality as a consequence of stock‐keeping issues that we observed for some time during Phase II. This did not compromise our conclusions because the final selection of target genes was based on an experimentally independent validation screen (see below).

### 3.2 Selection of genes for the validation screen

We wanted to define the *most effective target genes*, defined as those that lead to lethality most rapidly with minimal dsRNA exposure. For validation, we chose most of the genes that in the primary screen had shown 100% lethality and included many genes with 90% lethality (see Fig. S2 for more details on the selection). Specifically, from the 623 and 282 genes that had shown 100 or 90% lethality in the primary screen, respectively, 607 and 200 genes were included. In summary, 807 of 905 genes (further referred to as ‘*target genes*’) were included in the validation screen – they were tested in independent experiments using lower concentrations of dsRNA and monitoring the lethality over time. Additionally, 16 genes with lower lethality were tested to check for consistency with the results of the primary screen.

### 3.3 Validation screen to detect the most efficient RNAi target genes

**Figure 2:**
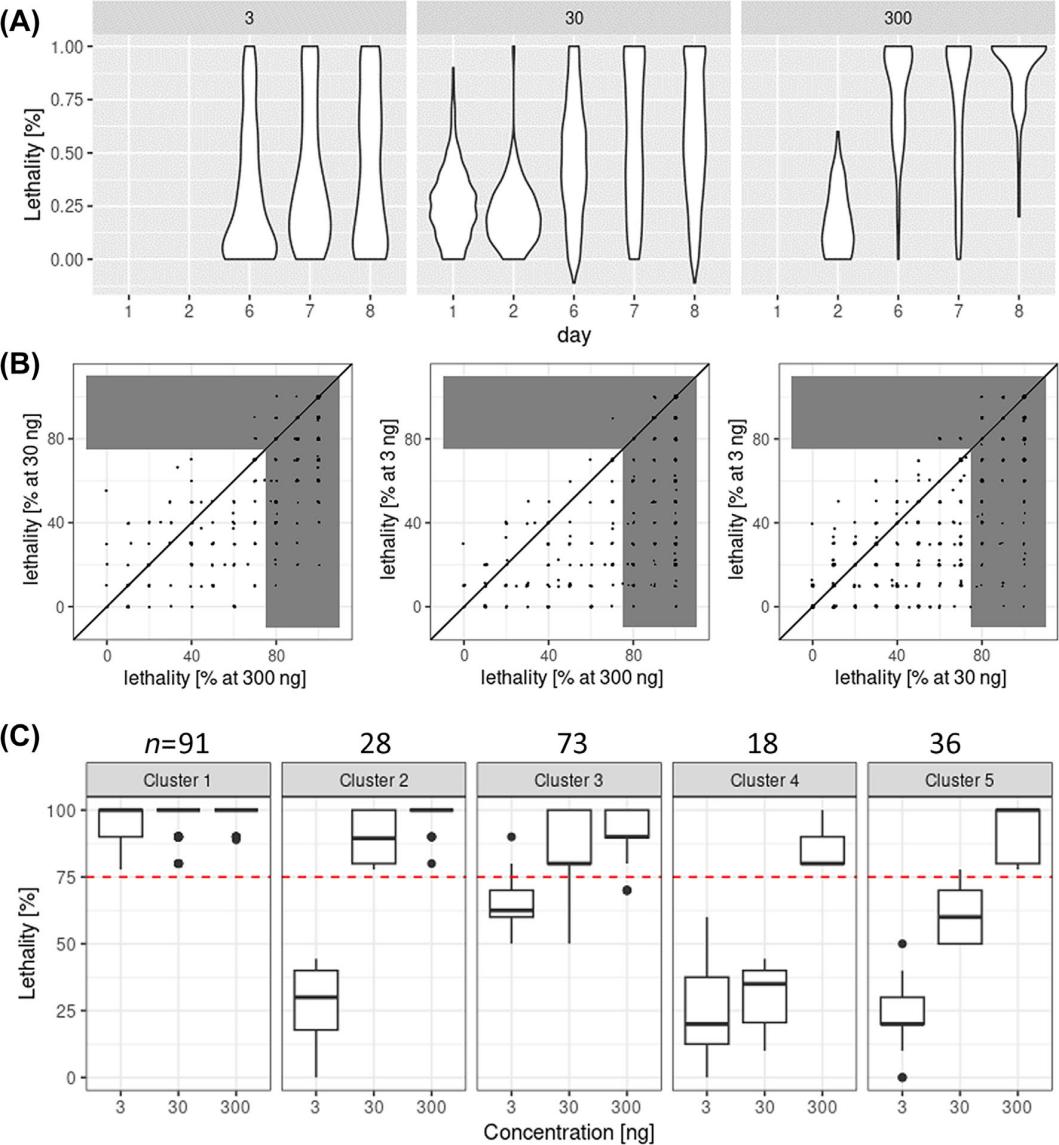
Distribution and correlation of lethality at different concentrations found in the validation screen. (A) The lethality distribution found in the validation screen is depicted along the time axis (in days postinjection; see Fig. S3 for separate documentation of the results of different validation screen phases). As expected, the effect is dose‐ and time‐dependent. Note that we assign lethality observed Day (D)1 and D2 postinjection to technical lethality. Notably, the distributions found with the lowest concentration (left panel) differ less along the time axis compared to higher concentrations. (B) Correlation of lethality between different concentrations of dsRNA targeting the same gene at D7 or D8 postinjection. Each dot reflects the lethality induced by a given dsRNA injected at the different concentrations (see axes). Lethality ≥75% is shaded in gray. As expected, the lethality increased with the concentration. The correlation of the two high concentrations (left panel) was high while the lowest concentration (3 ng μL^−1^) failed to induce lethality for a large number of genes, which did have strong effects at 30 or 300 ng μL^−1^ (see dots in right bottom part of gray area in the middle and right panels). Nevertheless, a number of genes induced lethality even at the lowest concentration (top right gray area). (C) Cluster analysis of a subset of the genes tested in the validation screen according to their level of lethality. Clusters 1–3 were used to define the *most effective target genes* used for subsequent analyses. See text for further details.

All 807 genes included in the validation screen were injected with a dsRNA concentration of 30 ng μL^−1^ and subsets were treated in addition with 3 or 300 ng μL^−1^ dsRNA. The lethality distributions revealed that 300 ng μL^−1^ closely reflected the results found in the primary screen (1 μg μL^−1^) in that a high degree of lethality of almost all tested genes was observed after 6–8 days [Fig. 2(A), right panel]. At 30 ng μL^−1^ concentration of injected dsRNA, the knock‐down led to lethality in many but not all genes [Fig. 2(A) middle panel]. The lowest concentration (3 ng μL^−1^) induced lethality in a yet smaller portion of genes [Fig. 2(A) left panel]. We concluded that the 300 ng μL^−1^ concentration was too high to be a stringent selection criterion, whereas 3 ng μL^−1^ was close to the lower limit of dsRNA concentration that can induce a lethal effect in *T*. *castaneum* by injection. While the lethality at the higher concentrations correlated [Fig. 2(B), left panel], many dsRNAs that induced lethality at higher concentrations failed to do so at 3 ng μL^−1^ [Fig. 2(B), middle and right panels].

### 3.4 Defining the most effective RNAi target genes

Based on our data, we considered two ways to define the *most effective target genes*. First, we used only the results from experiments with 30 ng μL^−1^ concentration because this had been used for all genes in the validation screen. From all these genes, 145 showed a lethality of 100% (0.9% of all genes of the primary screen) (see Table S3 for gene IDs). Although this approach included all genes from the validation screen, it did not take into account dose‐dependent responses and was therefore not used for follow‐up experiments. In a second approach, we performed cluster analysis on the subset of 246 genes that had been validated with 3 ng μL^−1^ and that had showed a lethality ≥75% at the 30 ng μL^−1^ concentration. Unsupervised k‐means clustering was performed based on the percentage lethality at different concentrations at D7 or D8 postinjection with dsRNA. We obtained five distinct clusters with Cluster 1 comprising the 91 most potent target genes with high lethality at all concentrations [Fig. 2(C)]. Cluster 3 (73 genes) comprised highly potent targets with some decline of efficiency at 3 ng μL^−1^ dsRNA injection whereas Cluster 2 (28 genes) showed more pronounced cutoff concentrations. Cluster 5 (36 genes) shows the clearest dose response and Cluster 4 (18 genes) lowest efficacy. See Table S4 for the gene IDs of these clusters, and Table S5 for respective GO‐term and KEGG analyses, and the top 15 GO terms of Cluster 1.

Taken together, our genome‐wide screen by injection in the red flour beetle revealed a list of 905 *target genes*, which represented an excellent basis for understanding biological processes targeted by these genes. The validation screen and cluster analysis led to the identification of 192 *most effective target genes* derived from clusters 1–3. These genes represent an excellent starting point for transfer to other pests.

### 3.5 The majority of the target genes are part of basic cellular processes

In order to reveal the biological processes and pathways, which should be targeted in RNAi‐mediated pest control, we asked which functions and pathways were enriched in the *very good target gene* set defined in our primary screen (905 genes representing 5.8% of all screened genes) compared with the set of genes with a mortality of ≤50%. The genes were annotated by similarity and functional domains using blast2go and mapped to KEGG pathways.^39^, ^41^ Gene ontology term enrichment analysis resulted in 393 enriched GO terms represented by at least five genes with *P* ≤ 0.01. The top GO terms according to their *P*‐value are listed in Table 1 (see Table S1 for all enriched GO terms; the IDs of the genes contributing to the top 15 GO annotations are found in Table S2). The top ‘GO terms biological process’ reflected predominantly basic cellular processes involved in gene expression such as translation, transcription and RNA metabolism (Table 1). Two terms were related to development but most of the underlying genes are involved in basic cellular processes that indirectly influence developmental pattern formation (see Table S2 for gene IDs). Notable exceptions are the nuclear hormone receptor Ftz‐F1 (TC002550) and the Glycogen synthase kinase‐3 beta (TC032822), which have direct functions in pattern formation in addition to metabolic processes.^42^ The GO terms of the domains ‘molecular function’ and ‘cellular component’ reflected those findings.

**Table 1:** Gene ontology enrichment of the set of very good RNAi target genes

Accession	Name	Ontology	Target genes	Total genes	*P*‐value
GO:0009792	Embryo development ending in birth or egg hatching	BP	88	228	3,15E‐38
GO:0006614	SRP‐dependent cotranslational protein targeting to membrane	BP	48	66	3,16E‐38
GO:0019083	Viral transcription	BP	43	60	6,86E‐34
GO:0000184	Nuclear‐transcribed mRNA catabolic process, nonsense‐mediated decay	BP	50	87	3,11E‐32
GO:0002119	Nematode larval development	BP	70	178	4,43E‐31
GO:0006413	Translational initiation	BP	47	82	2,77E‐30
GO:0002181	Cytoplasmic translation	BP	41	65	6,66E‐29
GO:0003735	Structural constituent of ribosome	MF	51	113	3,05E‐26
GO:0044822	RNA binding	MF	120	539	1,07E‐25
GO:0005840	Ribosome	CC	62	169	1,20E‐25
GO:0003729	mRNA binding	MF	61	164	1,27E‐25
GO:0009506	plasmodesma	CC	60	165	1,30E‐24
GO:0006364	rRNA processing	BP	44	95	2,35E‐23
GO:0005654	Nucleoplasm	CC	178	1097	4,55E‐21
GO:0005730	Nucleolus	CC	118	592	6,24E‐21

*Note*: The 905 genes with mortality ≥90% in the primary screen were compared to all genes with mortality ≤50% using hypergeometric distribution. The top 15 enriched gene ontology (GO) terms are shown. See Table S1 for all GO terms and Table S2 for respective gene IDs.

We asked whether signaling pathways would be good targets owing to their involvement in many biological processes. We found the NIK/NF‐kappa B, TNF, Wnt and MAPK signaling pathways to be significantly enriched in our set of candidate target genes, but they were not among the top 15 (see Table S2). Further GO terms associated with molecular functions comprised protein binding and cell–cell adhesion, as well as endocytosis and proton‐transporting ATPase activity. In line with the latter term, V‐ATPase had been introduced as a potent RNAi target gene before^4^ and has been successfully used by others since. The previously described enrichment of proteasome components in RNAi target genes^13^ was found in this genome‐wide dataset as well, albeit not with the highest scores. Other enriched GO terms in the cellular component domain included key cellular complexes such as the proteasome and ribosome, general compartments such as the vacuolar membrane, cytoplasm and nucleoplasm, and specialized structures such as the myelin sheath and exosome.

In order to map and visualize enriched GO terms and their functional interconnections, we created networks using REVIGO and GO slim annotations (Figs S4–S6).^40^ With respect to biological process, the network consists of two major subnetworks: one reflecting regulatory processes (top part in Fig. S4) and one reflecting transcription, translation and related decay processes (center part in Fig. S4). With respect to the category ‘cellular component’, only one network was found that included mainly translation and protein decay (Fig. S5).

In summary, our GO term analysis revealed that the set of *target genes* identified from the primary genome‐wide screen was highly enriched in basic cellular processes with translation, protein homeostasis, transcription and RNA biology being among the top processes. Our analysis shows that genes in biosynthetic processes are more abundant among the most efficient target genes than genes for structural components, which were not much enriched in our dataset.

In order to add pathway information, our *target genes* were assigned to KEGG pathways. Essentially, this analysis yielded pathways similar to those found by the GO term enrichment, for example ribosome, proteasome and spliceosome as top annotations, and a number of additional processes related to protein and RNA biology (Table 2). Interestingly, in this analysis the proteasome was recovered as one of the three top pathways reflecting our previous findings.^13^ Notably, the KEGG pathway ‘oxidative phosphorylation’ had a high score indicating that energy metabolism may be a good target for RNAi‐mediated pest control as well.

**Table 2:** Kyoto encyclopedia of genes and genomes (KEGG) enrichment of the set of target genes

Accession	Name	Good target genes	Total genes	*P*‐value	Good target genes (%)
ko03010	Ribosome	43	69	1,72E‐18	62.3
ko00190	Oxidative phosphorylation	30	58	2,50E‐10	51.7
ko03050	Proteasome	22	35	4,50E‐10	62.9
ko03040	Spliceosome	35	82	6,01E‐09	42.7
ko04145	Phagosome	24	52	2,81E‐07	46.2
ko04721	Synaptic vesicle cycle	16	30	2,69E‐06	53.3
ko03060	Protein export	11	16	3,54E‐06	68.8
ko03013	RNA transport	27	73	1,00E‐05	37.0
ko00970	Aminoacyl‐tRNA biosynthesis	12	25	0,00018991	48.0
ko03020	RNA polymerase	9	16	0,00027484	56.3
ko03022	Basal transcription factors	11	25	0,00089401	44.0
ko03015	mRNA surveillance pathway	16	46	0,00146617	34.8
ko03008	Ribosome biogenesis in eukaryotes	14	44	0,00716605	31.8
ko04623	Cytosolic DNA‐sensing pathway	7	16	0,0083228	43.8
ko04714	Thermogenesis	26	102	0,00923878	25.5

*Note*: The 905 genes with mortality ≥90% in the primary screen were analyzed for annotation in KEGG pathways.

### 3.6 Transfer to oral feeding in other pest species reveals 34 superior target genes

We tested some of the *most effective target genes* in other species for two reasons. First, our genome‐wide screen was based on injection of dsRNA, which probably has different characteristics to oral application. Unfortunately, oral application did not work for *T*. *castaneum* in our hands despite positive reports from others.^43^, ^44^, ^45^, ^46^ Second, limits of interspecies transferability had been observed previously.^13^, ^15^ Therefore, we tested for the transferability of *superior target genes* to different species and changing the delivery mode to oral application.

**Figure 3:**
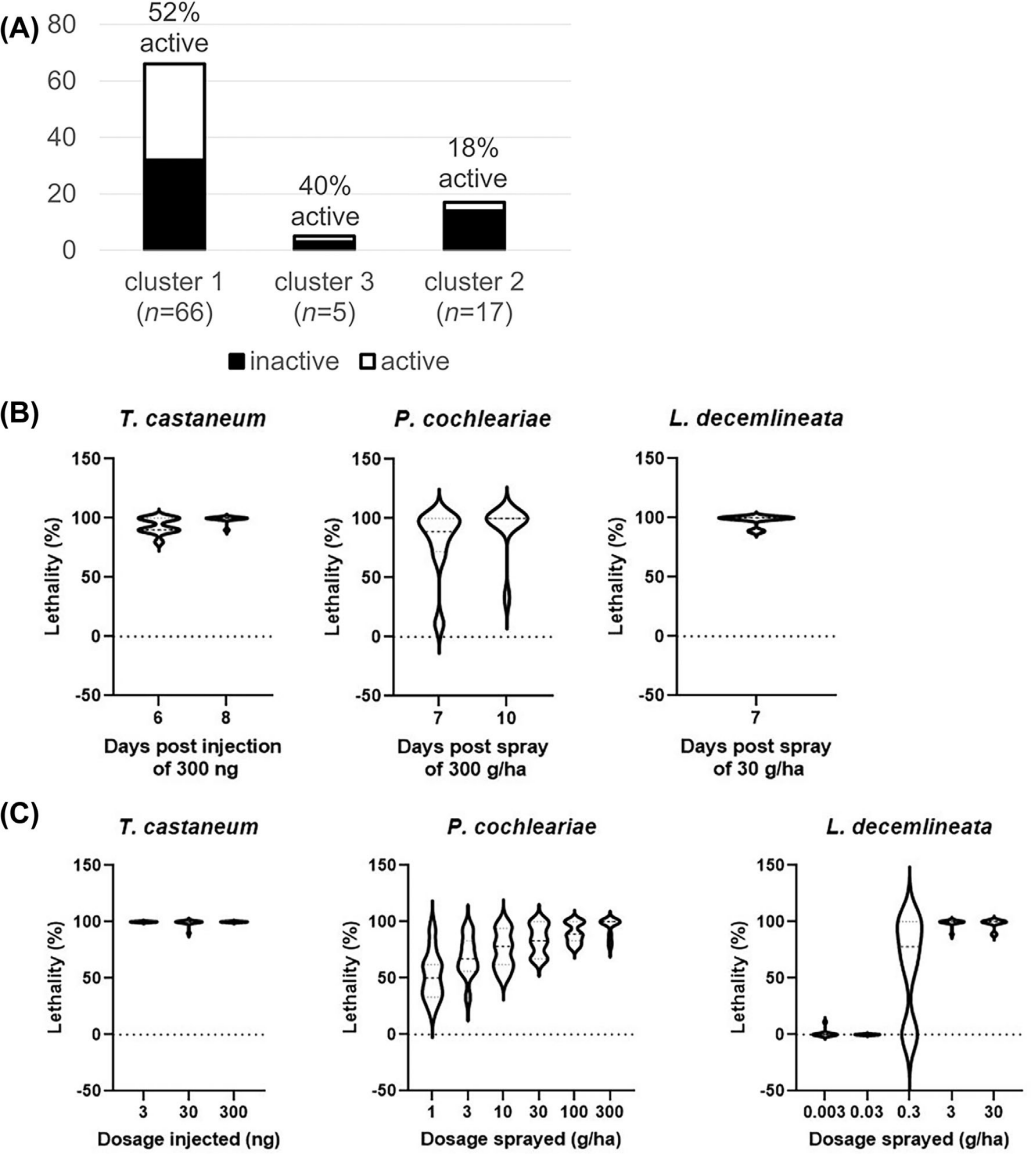
Transfer of most effective target genes to other species. (A) From 66 genes of Cluster 1, we found 52% to be transferable to *Phaedon cochleariae* by oral feeding. The transferability for the other clusters was lower. We defined the 34 genes transferred from Cluster 1 to represent our superior target gene set. (B) Time to mortality induced by 11 selected genes that were active in *Tribolium castaneum*, *P. cochleariae* and *Leptinotarsa decemlineata*. Lethality was found within 7–10 days in all three species. (C) These 11 genes were sensitive to small amounts of dsRNA but large species‐specific differences of sensitivity were observed.

We used the mustard beetle *Phaedon cochleariae*, a well‐described pest established for RNAi screening.^11^ We randomly chose 88 genes from our *most effective target genes* (i.e., clusters 1–3), determined their orthologs in *P*. *cochleariae* and tested respective dsRNAs by oral delivery. We used a concentration corresponding to 30 or 100 g ha^−1^ and checked for effects after 10 days maximum. We tested 66 sequences from Cluster 1 and found that 34 (52%) showed increased lethality by >50% compared to controls [Fig. 3(A)]. For Cluster 2 (17 genes) the rate declined to 18%, whereas for Cluster 3 (five genes) we found 40%. These results underscored the variability of the transfer across species and/or delivery modes. Furthermore, we defined the 34 successfully transferred genes from Cluster 1 to be our final selection of *superior target genes*. This set of genes represents a manageable number to be tested in other pest species yet may be sufficiently large to accommodate for species‐specific variability. The gene IDs of the 34 *superior target genes* are given in Table 3. Note that additional similarly effective target genes might be present, for example in the 26 nontested genes from clusters 1–3 or in the set of most efficient target genes that we defined by the alternative approach (see above). To confirm transferability of the *superior target genes*, we tested a subset of 12 genes in *L*. *decemlineata*, an organism that had shown excellent response to RNAi.^47^ Indeed, 11 of 12 sequences showed strong effects compared to our controls indicating a high degree of transferability (marked in gray in Table 3). Interestingly, these 11 genes led to lethality within 7–10 days in all tested species [Fig. 3(C)].

**Table 3:** Superior target genes recommended for transfer to other pest species

Tribolium gene ID	iBeetle number	Gene name
TC000069	iB_00011	Proteasome subunit beta type‐1
TC000614	iB_00141	Proteasome subunit alpha type‐6
TC000641	iB_00148	Coatomer subunit beta
TC002574	iB_00404	Signal recognition particle 54 kDa Short = SRP54
TC004425	iB_03754	Heat shock 70 kDa cognate 3
TC005185	iB_06598	Polyadenylate‐binding 1 Short = PABP‐1 Short = Poly(A)‐binding 1
TC005653	iB_07516	Snakeskin
TC006375	iB_04125	26S proteasome non‐ATPase regulatory subunit 6
TC006492	iB_04154	26S proteasome regulatory subunit 7
TC007891	iB_01280	26S proteasome non‐ATPase regulatory subunit 8
TC007999	iB_04411	26S proteasome regulatory subunit 6B
TC008617	iB_01375	Proteasome subunit beta type‐4
TC009191	iB_01493	transport Sec23A
TC009491	iB_01562	RNA‐binding 15
TC009675	iB_08675	26S proteasome regulatory subunit 4 Short = P26s4
TC009965	iB_04734	Ras‐related Rab‐1A
TC010003	iB_01640	DNA‐directed RNA polymerases I, II, and III subunit RPABC1
TC010318	iB_01665	Bifunctional 3‐phosphoadenosine 5‐phosphosulfate synthase (PAPS synthase)
TC010321	iB_04808	26S proteasome regulatory subunit 10B
TC010519	iB_01704	ATP‐binding cassette sub‐family E member 1
TC011058	iB_01793	Dynamin
TC011120	iB_01807	ROP
TC011182	iB_01820	60S ribosomal L7a
TC012303	iB_01965	Eukaryotic translation initiation factor 3 subunit A (eIF3a)
TC013782	iB_02204	Glycine–tRNA ligase
TC014413	iB_05628	ATP‐dependent RNA helicase WM6 Short = DEAD box UAP56 Short = Dmrnahel
TC014725	iB_09124	Prolactin regulatory element‐binding
TC015014	iB_02377	Clathrin heavy chain
TC015205	iB_08499	Proteasome subunit beta type‐3
TC015539	iB_09161	40S ribosomal S3a
TC031132	iB_07271	Pre‐mRNA‐splicing factor SYF1
TC033036	iB_02787	26S proteasome non‐ATPase regulatory subunit 1
TC034312	iB_09459	Splicing factor 3B subunit 1
TC034766	iB_01582	Tubulin beta‐3 chain

*Note*: These 34 genes were identified as most efficient target genes in *T*. *castaneum* by injection (i.e. they are part of Cluster 1) and were successfully transferred to *C*. *cochleariae* by feeding. The 11 genes shaded in gray were additionally successfully transferred to *L*. *decemlineata*.

## 4 DISCUSSION

### 4.1 RNAi‐mediated pest control can target biological processes not used by classic insecticides

With this work, we present the first genome‐wide screen of the most effective target genes and pathways. Our study provides a comprehensive whole genome view with respect to GO‐and KEGG pathways, even though about half of potential *superior target genes* were not tested owing to the necessity of restriction of effort (see Data S1 for discussion). Indeed, our unbiased approach revealed novel target pathways of which only some had been known previously from classic insecticidal processes such as synaptic vesicle cycle or oxidative phosphorylation. Most *target genes* acted in basic cellular processes such as transcription, protein translation, export and degradation. Notably, these processes are very different from the MoAs of classic insecticides. One reason might be that the proteins essential for basic cellular processes are often highly conserved between insects and vertebrates such that most chemical inhibitors would not pass the biosafety tests. Moreover, RNAi might be able to target the expression of genes whose protein products are not accessible for classic insecticides as a consequence of their cellular localization or quaternary structures known for instance from the proteasome or ribosomal proteins. To avoid cross‐effects on nontarget organisms, RNAi can be directed to diverged sequences including UTRs, which allows for species‐specific targeting of even highly conserved proteins.^10^, ^13^ Indeed, the pilot for this screen had identified the proteasome as prime target and the first sprayable application is based on a proteasome subunit.^9^, ^13^ In summary, RNAi opens essential basic cellular pathways for targeting, which have been protected from classic insecticides.

### 4.2 Comparison of our superior target genes to the target genes currently used

Our genome‐wide view allowed us to ask how far the previously used RNAi target genes were well‐selected. If so, they would be expected to be comprised in our lists. Importantly, several popular target genes such as chitin synthase, acetylcholinesterase or ecdysone receptor were found in none of our lists, indicating that there is room for improvement for respective applications by testing our *superior target genes*.

Three previously used genes were in our *superior target gene* list: Sec23, heat shock 70 kDa, and COPI coatomer β subunit (bold in Table 4) confirming that the respective previous screening efforts had identified excellent target genes. Notably, neither of the currently registered RNAi‐based products and only five of 12 commonly used target genes belonged to our top‐performing Cluster 1 genes (see Table 4). Another three targets represented different subunits of protein complexes that were targeted by one of our *superior target genes*: The proteasome, microtubules and the ribosome (gray in Table 4). While these genes are likely to be quite good target genes, testing our set of *superior target genes* would still be advisable as any increase of efficacy reduces the cost for application. The top 11 genes identified in a previous screen belonged to Cluster 1, whereas a machine‐learning approach to identify essential genes had revealed only one Cluster 1 target gene.^13^, ^48^

**Table 4:** Commonly used target genes compared to our gene sets

Target gene	Species/Reference	905 target genes	145 most effective target genes	91 Cluster 1 genes
Current products				
*Snf7* (SmartStax®PRO)	*D*. *virgifera virgifera* (Baum *et al*.,^4^)	Yes	Yes	No
*Proteasome Subunit Beta Type‐5* (Calantha™)	*L*. *decemlineata* (Rodrigues *et al*.^9^)	Yes	No	No
Commonly targeted genes				
*V‐ATPase* subunits	*D*. *virgifera virgifera* (Baum *et al*.,^4^)	Yes	Yes	Yes
*α‐tubulin*	*D*. *virgifera virgifera* (Baum *et al*.,^4^)	No	No	No
*β‐Actin*	*L*. *decemlineata* (Zhang *et al*.^7^)	No	No	No
Smooth septate junction (SSJ)	*D*. *virgifera virgifera* (Hu *et al*.,^49^)	Yes	Yes	Yes
***Heat shock protein 70***	*Agrilus planipennis* (Rodrigues *et al*.,^50^)	Yes	Yes	Yes
***Sec23***	*L*. *decemlineata* (Zhu *et al*.,^47^)	Yes	Yes	Yes
*Inhibitors of apoptosis*	*Aedes aegypt*i (Pridgeon *et al*.^51^)	No	No	No
***COPI coatomer β subunit***	*D*. *virgifera virgifera* (Baum *et al*.,^4^)	Yes	Yes	Yes
*Ribosomal protein L19*	*D*. *virgifera virgifera* (Baum *et al*.,^4^)	Yes	No	No
*Chitin synthase*	*Spodoptera exigua* (Tian *et al*.,^52^)	No	No	No
*Acetylcholinesterase*	*Helicoverpa armigera* (Kumar *et al*.,^53^)	No	No	No
*Ecdysone receptor*	*Nilaparvata lugens* (Yu *et al*.,^54^)	No	No	No
Previous large‐scale RNAi screen				
**All top 11 genes**	*T*. *castaneum* (Ulrich *et al*.^13^)	Yes	Yes	Yes
*Prediction based on machine learning*				
*ATP‐dependent RNA helicase spindle‐E‐like*	*T*. *castaneum* (Beder *et al*.^48^)	Yes	No	No
*ATP‐dependent RNA helicase abstrakt‐like*	*T*. *castaneum* (Beder *et al*.^48^)	Yes	No	No
*Eukaryotic translation initiation factor 3 a*	*T*. *castaneum* (Beder *et al*.^48^)	Yes	Yes	Yes
*ATP‐dependent RNA helicase Dbp45A‐like*	*T*. *castaneum* (Beder *et al*.^48^)	No	No	No
*ATPase family AAA domain‐containing protein 3‐like*	*T*. *castaneum* (Beder *et al*.^48^)	No	No	No

*Note*: Selected target genes were checked whether they were included in the 905 *target genes* (identified in the primary screen), the 145 *most effective target genes* (based on the validation screen based on 30 ng μL^−1^; see above) or the 91 genes from Cluster 1. Three genes previously published by others were included in our *superior target gene* list (shown in bold) and three targeted different subunits of the protein complexes targeted by *superior target genes* (shaded in gray).

A limitation of our approach was that the primary screen in *T*. *castaneum* was injection‐based, whereas pest control strategies typically aim for oral dsRNA delivery. However, the tissues primarily impacted by oral dsRNA delivery, such as the gut, also were covered in our study owing to the robustness of systemic RNAi in *T*. *castaneum* upon dsRNA injection. Hence, our primary screen had the potential to identify thosee targets effective through oral dsRNA delivery (i.e. not many false negatives). However, our injection‐based screen also is likely to have identified targets that would be challenging to knock down through oral delivery, making them unsuitable for pest control (i.e. false positives). In order to exclude targets that do not perform by oral application, we added the transfer work in *P*. *cochleariae* using oral dsRNA delivery. This showed that ≈50% of the most effective targets in *T*. *castaneum* (Cluster 1) also were effective in *P*. *cochleariae*. Thus, both the mode of dsRNA delivery and the change in species are likely to have affected the transferability of effective genes. This transfer work enabled filtering out genes that might be less suitable for pest control as well as those less likely to be transferred from one species to another. Indeed, when the target genes transferable to *P*. *cochleariae* (superior target genes) were tested in *L*. *decemlineata*, we observed almost complete transferability (11 of 12 tested genes were effective; see Table 3), suggesting that these superior target genes also may be highly transferable to other insect pests, especially coleopterans.

In summary, our superior target genes perform better than most genes previously selected based on knowledge on protein functions – at least under the conditions and species used in this study. One reason for the power of unbiased screening could be that the knowledge‐based approach does not take into account additional parameters that influence the RNAi response such as protein stability, compensatory pathways or paralogs, or compensatory upregulation of expression. Given the lack of knowledge of most parameters for most of the genes, our unbiased large‐scale screen seemed the tool of choice for the identification of the most efficient target genes. Interestingly, both current RNAi‐based commercial products are targeting genes that comprised our *very good target gene* set (905 genes with >90% lethality) and one of them, Snf7, even belonged to the *most effective genes*. This highlights the potential for transferability from an unbiased screen in a model species to the market.

### 4.3 Suggestion for detecting target genes in other pests

Three key findings influence our suggestion on the identification of the best target genes in additional pest species (see Cedden and Bucher^55^ for extensive discussion). First, this work shows that the different MoA of RNAi leads to a different set of target genes compared to classic insecticides. The respective genes and pathways may therefore be suboptimal targets. Second, past efforts of many laboratories have shown that target genes identified in one species also are often usable in other species. However, no consensus has emerged on ‘the best target gene’ and a systematic literature search has actually suggested that the efficiency observed for a given target gene is likely to be influenced by species‐specific variabilities, which seem to be unpredictable.^15^ This suggests that a search for the most effective target genes should start with a small screen including several putative target genes. Third, in this work, we have identified a set of 34 *superior target genes*, which have been effective in two species and 11 target genes, which have been effective even in three beetle species. We consider these sets of genes excellent starting points for identifying efficient targets. For a comprehensive discussion of our thoughts on the reasons for the species‐specific variability and our view on the best approach to detect target genes, see Cedden and Bucher^55^.

## 5 CONCLUSIONS

Our genome‐wide approach revealed the processes and genes that are the best targets for RNAi‐based pest control of coleopteran pests – predominantly highly conserved genes acting in basic cellular and biosynthetic processes. Furthermore, our short list of *superior target genes* provides an excellent starting point for identifying very effective targets in other insect pests and may even be transferrable to more distant taxa (see Text S2 for more details). While it may seem unlikely to identify more efficient protein‐coding target genes, it remains elusive how well noncoding RNA targets may perform. Another future challenge will be to increase the efficacy of the specific target sequence by rational design.

## CONFLICT OF INTEREST

BB, RN, and SG are employees of the Crop Science Division of the Bayer AG, which largely funded the project. All results are shown without restriction. A patent application for selected sequences was discontinued.

## Supporting information


**Data S1.** Supporting Information.

**Table S1.** Gene ontology (GO) terms and Kyoto encyclopedia of genes and genomes (KEGG) pathways enriched in very good target genes.

**Table S2.** Gene lists underlying the top 15 gene ontology (GO) terms.

**Table S3.** *Most effective target genes* based on lethality at 30 ng μL^−1^.

**Table S4.** *Most effective target genes* based on clustering.

**Table S5.** Gene ontology (GO) and Kyoto encyclopedia of genes and genomes (KEGG) enrichment analysis of clusters 1–5.

**Table S6.** Lethality results of the primary screen for all genes.

## Data Availability

The data that support the findings of this study are openly available in iBeetle‐base at https://ibeetle-base.uni-goettingen.de/.
