# Supplementary material for: Superior target genes and pathways for RNAi‐mediated pest control revealed by genome‐wide analysis in the beetle Tribolium castaneum
Source: Pest Manag Sci. 2024 Nov 5;81(2):1026–36. doi: 10.1002/ps.8505 (PMC11716340; doi:10.1002/ps.8505)
Supplement: Supplementary file 1 — Data S1. Supporting Information. [file PS-81-1026-s007.pdf]

Superior target genes and pathways for RNAi mediated pest control revealed by genome wide analysis in the beetle *Tribolium castaneum*

Benjamin Buer[1]*; Jürgen Dönitz[2/3]*; Martin Milner[2]*; Sonja Mehlhorn[1/2]; Claudia Hinners[2]; Janna Siemanowski-Hrach[2]; Julia K. Ulrich[1]; Daniela Großmann[2/3]; Doga Cedden[2]; Ralf Nauen[1]; Sven Geibel[1]#; Gregor Bucher[2]#

[1]Crop Science Division, Bayer AG, R&D, Pest Control, Monheim, Germany

[2] University of Göttingen, Johann-Friedrich-Blumenbach Institute, GZMB, Department of Evolutionary Developmental Genetics, Göttingen, Germany

[3] University Medical Center Göttingen, Department of Medical Bioinformatics, Göttingen, Germany

Supporting Information

Contents

1. Supporting Text 2

1. How comprehensive was our analysis? 2

2. Considerations for the identification of the most effective target genes for RNAi mediated pest control 2

Supporting figures 4

Supporting figure 1: Schedule and procedure for the high-throughput primary screen........ 4

Supporting Figure 2: Lethality distribution of the primary screen as basis for the selection for the validation screen 5

Supporting Figure 3: Lethality distribution of the validation screen 6

Supporting Figure 4: Revigo network of GO terms “biological process” 7

Supporting Figure 5 Revigo network of GO terms “cellular component” 8

Supporting Figure 6: Revigo network of GO terms “molecular function” 9

1. Supporting Text

1. How comprehensive was our analysis?

The primary screen (15.530 genes) covered 93,6 % of the current protein coding gene set of the *T. castaneum* genome assembly OGS3 [46] and mainly missed genes that could not be cloned from cDNA and genes that were affected by the usual loss of experiments during high throughput screens (see Supplementary Table 6 for all results of the primary screen). Therefore, our list of 905 *target genes* (top 5.8 % of the tested genes) is very comprehensive and our conclusions on GO terms and KEGG pathways provide the first and a very robust genome-wide view on that matter. Likewise, the validation screen was quite comprehensive where 807 out of 905 *target genes* were tested (89,2%).

The subsequent steps had the aim of identifying a manageable number of superior target genes for transfer to other pests rather than providing comprehensive analyses. Therefore, the cluster analysis was based only on those 443 genes, which had been validated with the lowest concentration (54,9 % of 807 genes in the validation screen). Due to this restriction, about half of the genes matching our criteria for *superior target genes* are probably missing from our list. Likewise, we tested a subset of 66 out of 91 genes from Cluster 1 (72,5 %) to define our list of *superior target genes*. This means that another dozen genes or so from Cluster 1 may show a similar efficacy when transferred to other species. Given the high transferability of our *superior target genes* to another pest species (11 out of 12; 91,7 %) we think that 34 genes are a sufficient and at the same time manageable number. If testing of the entire *superior gene* list does not result in an efficient RNAi response in an organism, the root cause is likely to lie in other reasons than the selection of the appropriate target gene.

2. Considerations for the identification of the most effective target genes for RNAi mediated pest control

We propose keeping the following considerations in mind when planning to identify the target genes for RNAi mediated pest control:

First, the selection of a target gene based on the knowledge of its essential function is often suboptimal because we lack the knowledge of all the other parameters influencing an efficient RNAi response such as protein stability, compensatory reactions, the developmental stage sensitive to that gene, dsRNA stability etc. Notably, some excellent targets for chemical insecticides have performed poorly when targeted by RNAi.

Second, due to species-specific variation of parameters influencing the effect of gene function and amenability to RNAi, a gene with an excellent response in one species may be less effective in another. Hence, there is no such thing as *the one best target gene*. See table in Mehlhorn et al. 2021b for examples. In practice, several candidate target genes have to be tested rather than relying on the selection of one.

Third, our list of *superior target genes* is an excellent starting point for a small scale screen to identify the best targets in another species. Such an approach focuses on some of the most promising targets but it still considers the possibility of species-specific variability. Testing 34 genes will be realistic for most systems and we consider the likelihood to be rather high that at least one of them will belong to the top group in the given species. If a more comprehensive approach is wanted, the remaining genes of Cluster 1 could be included or the 145 genes of the *most effective target genes* could be tested.

Fourth, careful controls and independent replicates are paramount to avoid false-positive reports on RNAi in pest control. Lethality is a very unspecific phenotype that is often elicited by a variety of technical variables such as contaminated injection needles, poor dsRNA preparations, stock keeping issues, infection status etc. Before testing RNAi for pest control with such an unspecific readout, an efficient RNAi response should first be confirmed. To that end, the use of non-lethal target genes with a clear phenotypic readout such as pigmentation genes are advisable. See Mehlhorn et al. 2021b for suggestions.

Fifth, many of the target processes are highly conserved in eukaryotes. Hence, they might be valuable targets in other economically relevant arthropods such as spider mites or even other clades of eukaryotes such as fungi.

Supporting figures

Supporting figure 1: Schedule and procedure for the high-throughput primary screen.

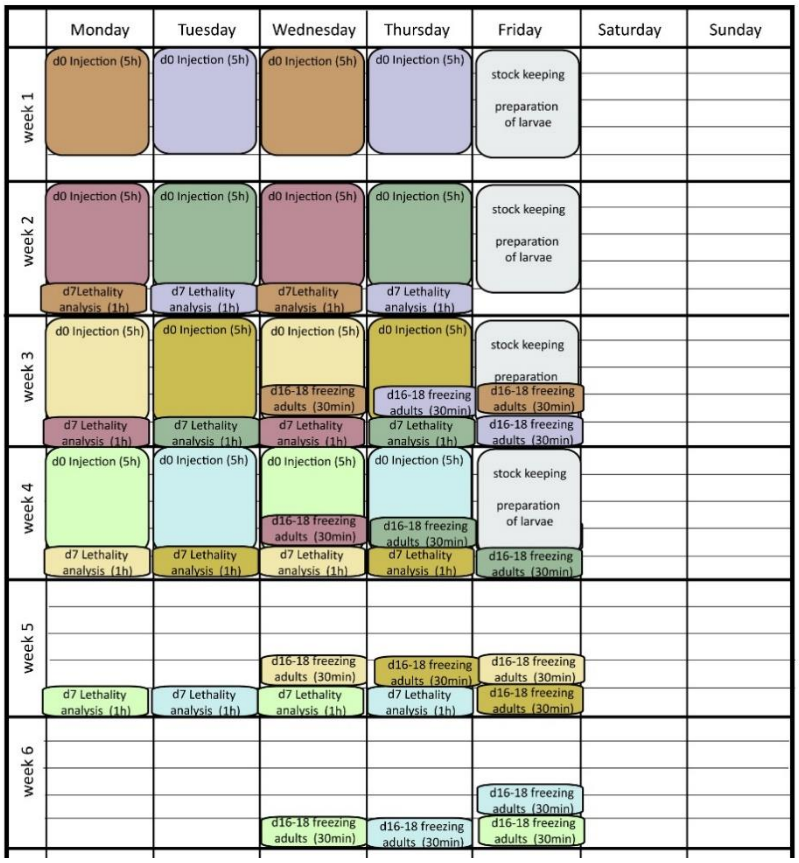

Schedule depicting the different processing steps - one set of parallel experiments is shown in the same color. In this depiction, 16 such parallel experiments are shown (i.e. 4 per week). In the primary screen, dsRNAs at a concentration of 1 µg/µl were injected into 10 larvae per gene (stages L5 or L6).

On each injection day (d0), dsRNAs targeting 39 different genes plus one buffer control were injected into 10 larvae of L6 each (L5 used only in case of need). Injection was done four days a week (Monday-Thursday) while the fifth day was used for stock keeping and collection of larvae for the following week's injections. The first injection of each day was a buffer control, which served as negative control and was at the same time needed as warm-up for the injection procedure (We had previously observed a generally increased level of background lethality in the first injection of a day).

Seven days later (d7) the survival was scored for the first time while 16-18 days later survival was checked a second time (data not shown) and the pupae/adults were frozen for later morphological analyses.

The schedules were interleaved such that week 5 of the first round of experiments is at the same time week 1 of the following repetition of the schedule (not shown). This procedure was performed by one person for approximately two years showing that the workload of screening 156 genes per week (plus controls) was sustainable. We would consider it challenging to increase the throughput if the screener had to do it over extended time considering European worker protection standards.

Supporting Figure 2: Lethality distribution of the primary screen as basis for the selection for the validation screen

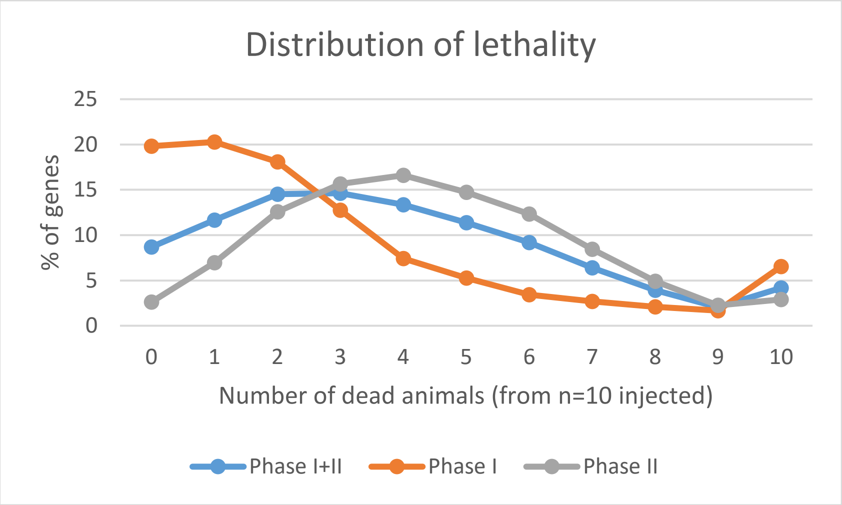

In order to determine the cutoff for selecting target genes for the validation screen, we used the distribution of dead animals per experiment for the earliest available lethality data for phase I (11 days post injection, orange line), phase II (7 days post injection grey line) and combined those datasets (blue line). We expected a distribution of technical or background lethality in addition to the gene specific RNAi effects. Indeed, we found that the lethality distribution of all datasets approached a minimum at 90 % dead animals (9 out of 10 injected) while the value for 100 % lethality was higher again. This increase of the 100 % value is best explained by RNAi induced lethality in those datasets. The number of experiments with a 90 % value was not clearly increased and therefore likely contained a portion of false positive datasets, i.e. data where technical lethality had contributed to the signal. Hence, for the validation screen we considered all genes showing 100 % lethality as candidates but still included some genes with 90 % lethality in order not to miss good candidates.

Supporting Figure 3: Lethality distribution of the validation screen

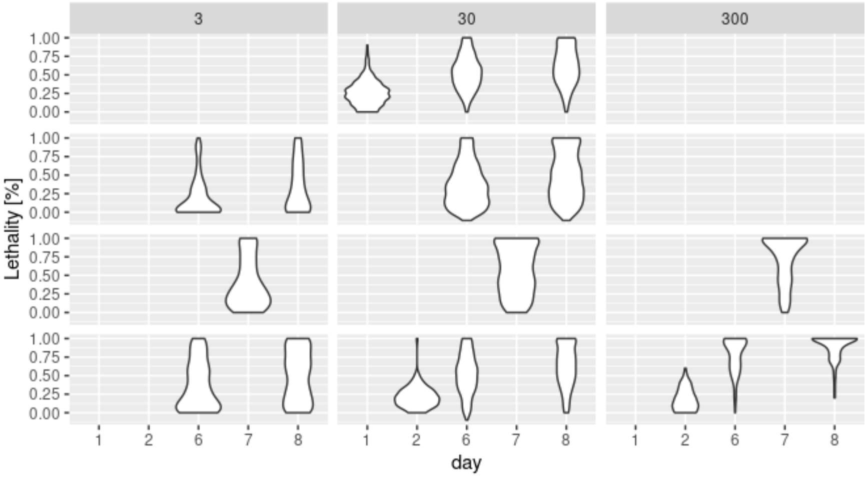

The validation screen (total n=843 genes) was performed in different phases by different people and under changing organizational constraints. In the first and second part, all three concentrations were used (bottom two rows). As the higher concentration seemed to reflect the results of the primary screen, that concentration was skipped in the subsequent phases of the validation screen (two top rows). The last part of the validation screen was performed only for the 30 ng/ul concentration (top row; n=400), which had shown to relieably separate very good from less efficacious target genes.

Supporting Figure 4: Revigo network of GO terms "biological process"

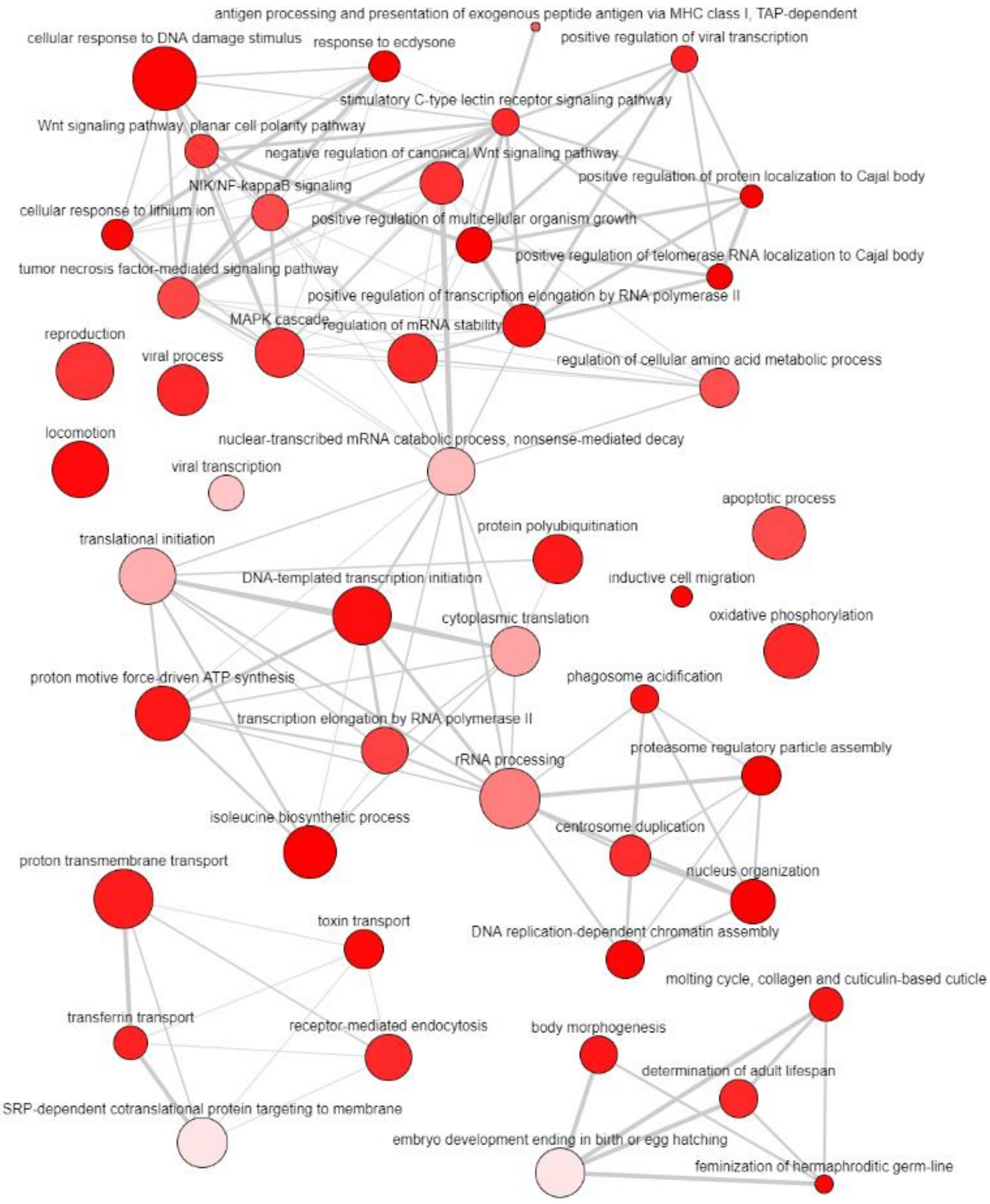

The color of the bubble corresponds to the p-value as given in the Supplementary Table 1: More intense colors reflects higher confidence. The size of the bubble corresponds to the LogSize p-value for the GO Term.

Supporting Figure 5 Revigo network of GO terms “cellular component”

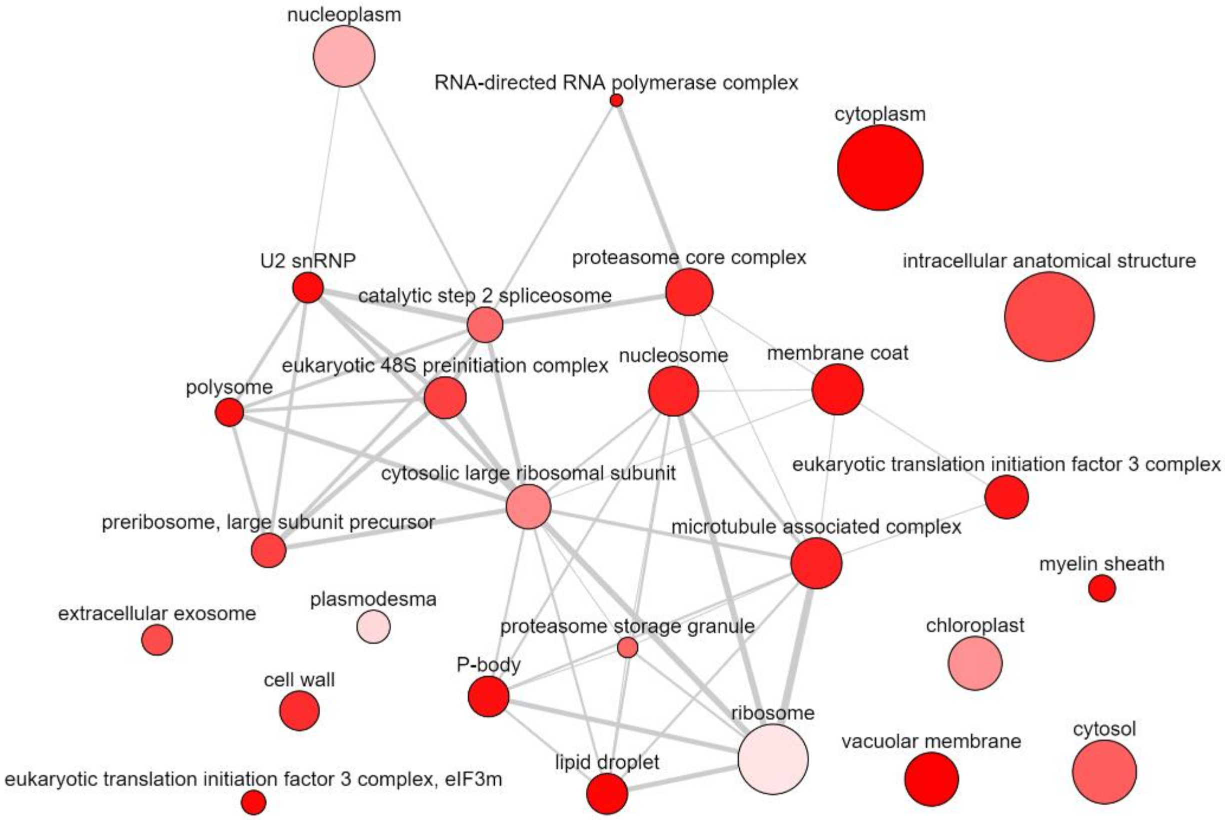

The color of the bubble corresponds to the p-value as given in the Supplementary Table 1: More intense colors reflects higher confidence. The size of the bubble corresponds to the LogSize p-value for the GO Term.

Supporting Figure 6: Revigo network of GO terms “molecular function”

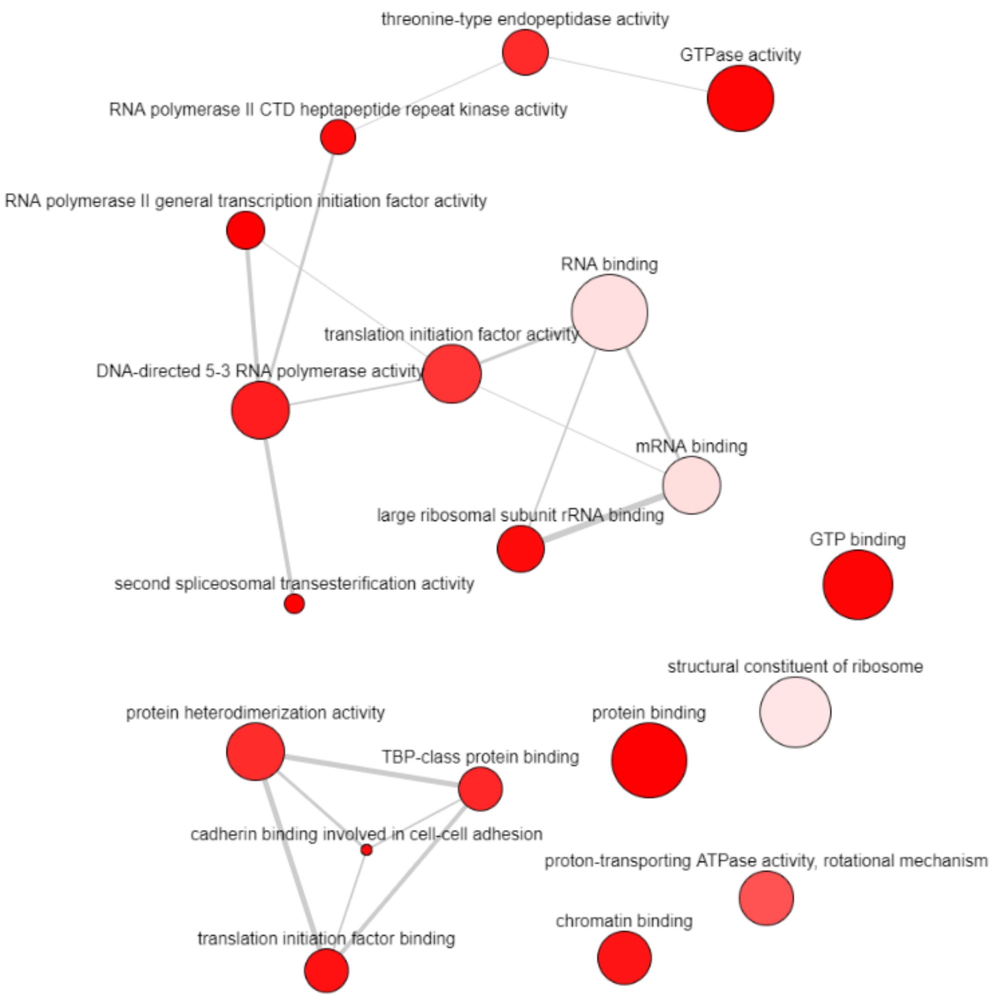

The color of the bubble corresponds to the p-value as given in the Supplementary Table 1: More intense colors reflects higher confidence. The size of the bubble corresponds to the LogSize p-value for the GO Term.
